# Molecular basis for protein–protein interactions

**DOI:** 10.3762/bjoc.17.1

**Published:** 2021-01-04

**Authors:** Brandon Charles Seychell, Tobias Beck

**Affiliations:** 1 Universität Hamburg, Department of Chemistry, Institute of Physical Chemistry, Grindelallee 117, 20146 Hamburg, Germany; 2 The Hamburg Centre for Ultrafast Imaging, Hamburg, Germany

**Keywords:** characterisation methods, heterooligomeric complex, homooligomeric complex, molecular interactions, protein–protein interactions

## Abstract

This minireview provides an overview on the current knowledge of protein–protein interactions, common characterisation methods to characterise them, and their role in protein complex formation with some examples. A deep understanding of protein–protein interactions and their molecular interactions is important for a number of applications, including drug design. Protein–protein interactions and their discovery are thus an interesting avenue for understanding how protein complexes, which make up the majority of proteins, work.

## Introduction

From signalling over transport to catalysis, the broad functionality of proteins is essential in the cellular machinery. To this effect, proteins can be seen as the workforce of the cell. Proteins relay some of their functionality via interactions between protein nodes called protein–protein interactions (PPIs). Hedin characterised the first PPI with trypsin and antitrypsin in 1906 [1], which provided a landmark for the awareness of what role PPIs have in cellular physiology. In fact, even though individual proteins perform essential functions, their effectiveness in the cell can only be fully exploited via interactions, either in the form of PPIs or with other metabolites and biomolecules, such as nucleic acids. Thus, the identification of the molecular binding partners that proteins interact with is an interesting avenue to facilitate the discovery of the protein functionality and the corresponding pathways. Important roles of PPIs include hormone reception [2], protease inhibition [3], antibody–antigen complexes [4], gene regulation [5], and large biomolecular assemblies [6]. PPI identification and prediction are important for targeting anticancer strategies [7], therapeutic interventions [8], and are crucial for potential drug discoveries [9]. This minireview will give a short insight into the different characterisation methods to characterise PPIs. Moreover, it will provide an examination of the different molecular interactions of PPIs and their role in protein–protein complex formation. Finally, we will give a brief account of some examples for higher-order protein complexes and the PPIs involved.

## Review

### Characterisation methods for PPIs

**Figure 1:**
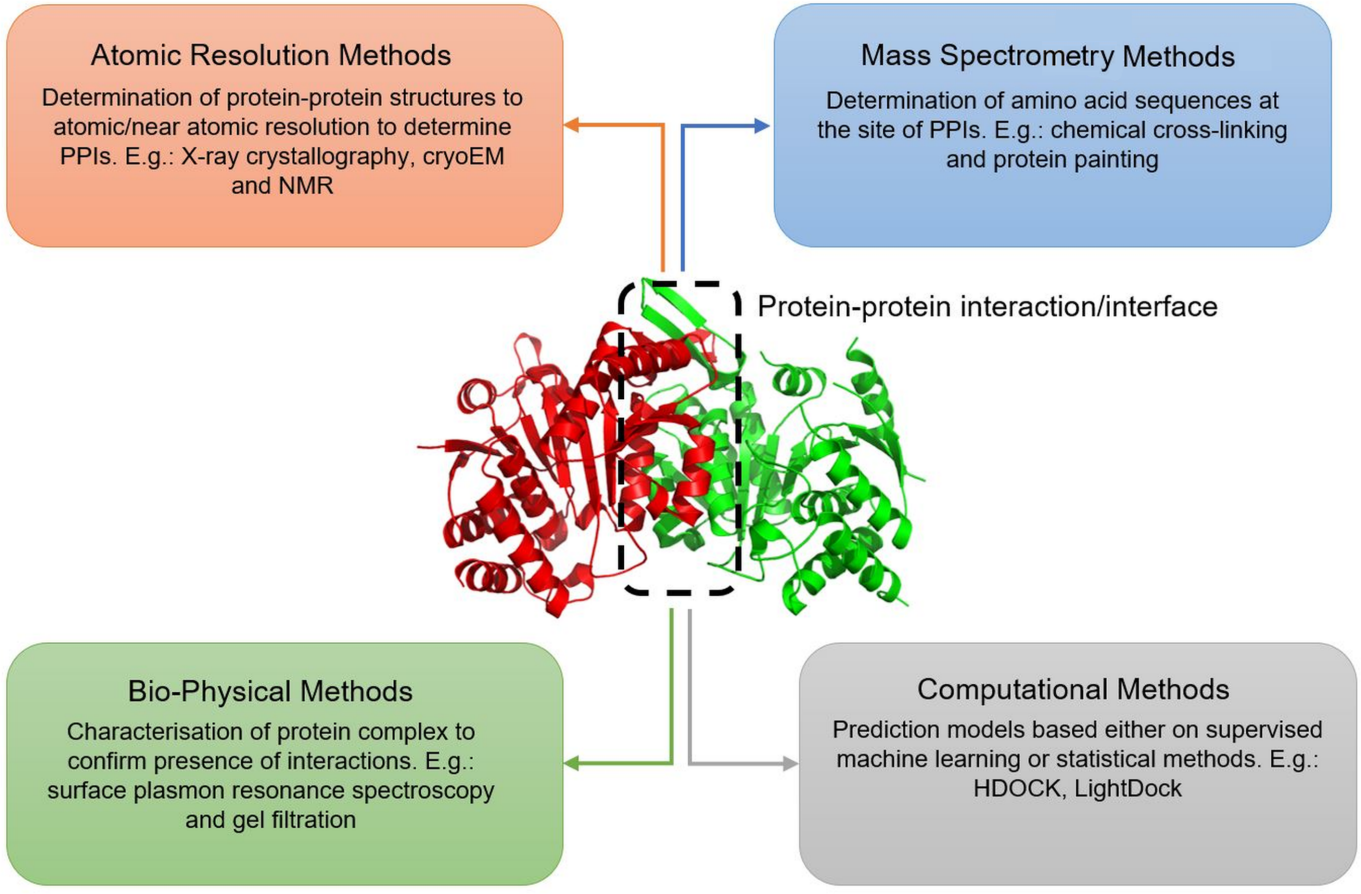
Different methodologies employed to study PPIs. The protein used for illustration is the heterodimer of human NAD-dependent isocitrate dehydrogenase (Protein Database (PDB): 6KDF) [10]. Figure adapted from Carter et al. [11].

Particular PPIs can be relatively difficult to study since in vivo*,* any particular protein is present amongst a plethora of proteins and other biological molecules, all of which have their own biological role and chemical interactions. Characterisation methods that can be employed to study PPIs can be divided into four categories: atomic resolution methods, mass spectrometry methods, biophysical methods, and computational methods. These methods are summarised in Figure 1.

Atomic resolution methods focus on the structure determination of protein complexes and elucidating the PPIs at a near-atomic resolution. Such methods include X-ray crystallography [12], nuclear magnetic resonance (NMR) [13], and cryogenic electron microscopy (cryo-EM) [14]. Traditionally, X-ray crystallography was the preferred method to solve the protein structure and determine protein–protein interfaces. However, protein crystallography has the limitation that some protein assemblies have a low diffraction quality and are difficult to crystallise [15]. In the past few years, advances in the cryo-EM technology attracted the interest of more and more structural biologists. In fact, the number of cryo-EM structures has steadily been increasing over the recent years, with over 12,500 electron density maps being deposited in the Electron Microscopy Data Bank [16]. The main advantage that cryo-EM has compared to X-ray crystallography is that the former does not require crystals, thus making it easier or sometimes even possible at all to study flexible protein assemblies that do not crystallise. Albeit this, the main limitations of cryo-EM included a lower resolution of protein structures and size requirements of the proteins under investigation. Nonetheless, resolution and size barriers are continuously being broken, with resolutions of up to 1.15 Å (human apoferritin, EMD-11668, PDB: 7a6a [17]) and structures as small as 25 kDa (*Bacillus subtilis* 50S subunit-nascent chain-tRNA complex, EMD-4799, no available PDB [18]) were solved. Protein crystals can also be used in conjunction with cryo-EM, employing a technique known as microcrystal electron diffraction (MicroED). With this method, thin 3D crystal slices are used to obtain the protein (or any other organic molecule [19]) structure. A resolution of up to 0.6 Å (RNA-binding protein FUS, residues 37–42, EMD-0699, PDB: 6KJ4 [20]) were obtained when using this method. Nannenga and Gonen gave a detailed account on MicroED [21].

As another category, mass spectrometry methods are used to determine the amino acid sequences at the PPIs site. Examples include protein painting [22] and chemical cross-linking [23], hydrogen–deuterium exchange mass spectrometry (HDXMS) [24], and fast photochemical oxidation of proteins [25]. In protein painting, small-molecular dyes are introduced to protein complexes where the dyes bind non-covalently to solvent-accessible surfaces. The protein–protein interface is not solvent-accessible, and thus the dye molecules do not bind to this region. The protein complex is first “painted” with the dyes and digested using proteases, such as trypsin. Different proteases provide different specificities, and thereby enabling different protein regions to be studied. Due to the dye molecules, dye-masked regions cannot be digested by the protease, leaving only the protein–protein interface and the solvent-inaccessible regions to be digested and detected by a mass spectrometer. Separately, the proteins that compose the complex are dyed, digested, and detected. The fragments that are detected in the painted complex but not detected in the painted protein monomers are the regions that were solvent accessible in the monomers but became inaccessible due to the complex formation, i.e., the protein–protein interface. Limitations of this approach include the possibility of having no protease cleavage regions in the interface region and the size of the probes used [22]. These limitations can be overcome by using HDXMS. Even though HDX dates back to the 1950s [26], advances in data analysis software and automation in liquid chromatography–mass spectrometry over the past decade made HDXMS an increasingly attractive tool for biochemists. In HDXMS, changes in the mass associated with the isotopic exchange between amide hydrogen atoms and surrounding deuterated solvent are measured. The incorporated deuterium can then be localised through protease-generated peptide fragments or fragmentation within the mass spectrometer. The rate of hydrogen-to-deuterium exchange provides insight into the solvent accessibility. HDXMS provides information on the conformation of the protein, PPIs, and even protein–ligand interactions and conformational changes within a protein complex. In addition to this, HDXMS has the advantage of using a low concentration of proteins (in the high-nM or low-µM range) and providing higher-resolution data on the solvent accessibility, compared to probes used in protein painting. However, even though HDXMS is a very reproducible and straightforward method (albeit the experiment has to be carefully and properly performed), data analysis, even with recent software automation, is the major limitation as the interpretation requires a certain level of expertise.

Biophysical methods are used to characterise the protein complex and to confirm the presence of certain interactions. Biophysical approaches are important to determine binding affinities, enthalpy changes, entropy changes, and the on- and off-rates of binding, amongst others. Examples include surface plasmon resonance (SPR) spectroscopy [27–28], gel filtration [29], isothermal titration calorimetry (ITC) [30–31], fluorescence resonance energy transfer [32], and microscale thermophoresis [33]. In SPR spectroscopy, one of the protein molecules that make up the complex is first immobilised on a metal surface. The orientation can be controlled by using, for example, an anti-affinity tag antibody [34] or an NTA surface [35]. Binding to the surface increases the density and the refractive index. Polarised light is irradiated on the surface at an angle around which SPR occurs (resonance angle). The binding partner is subsequently passed over the immobilised protein, and the metal surface is irradiated at the same angle. If the partner protein binds with the immobilised one, a shift in the SPR angle occurs, indicating that PPIs have taken place, and the binding affinity, measured as the dissociation constant *K*_d_, can be calculated [36]. Another commonly used biophysical method is ITC. In ITC, the binding partner is mixed with the protein solution at various concentrations, and the heat released or absorbed as the proteins (or the protein and the ligand) interact is measured. Since the measurements are proportional to the concentration of the analytes, it is imperative that accurate measurements are used. ITC, in contrast to SPR, has the advantage of measuring the affinity in solution (and not in an immobilised form). Moreover, with the use of high-sensitivity equipment, ITC is used to determine an array of thermodynamic properties, including the binding constant *K*_b_, the reaction stoichiometry (*n*), the observed molar calorimetric enthalpy Δ*H*_obs_, the entropy Δ*S*, the heat capacity of the binding Δ*C*_p,obs_, and the change in the Gibbs free energy Δ*G*. As a result, ITC is used to provide a complete thermodynamic characterisation of the binding reaction.

Computational methods are used to predict PPIs and interfaces. The advantage of performing in silico experiments includes narrowing down the number of the binding partners to be tested in vitro or in vivo. Computational methods include supervised machine learning, where models are predicted from positive and negative training datasets [37], and statistical methods using genome-based data to predict interactions [38]. However, some PPIs can be difficult to predict using computational programmes, and thus resulting in absent or wrong PPIs.

It is important to note that a full characteristic profile of PPIs can only be achieved when the aforementioned methodologies are used in conjunction with each other. For example, computational methodologies can be the initial starting point to predict and study PPIs, thereby reducing the project laboratory workforce and costs. The validation of PPIs can then be determined by using HDXMS together with cryoEM. The full thermodynamic profile can subsequently be determined using biophysical methods, yielding the full picture of a particular PPI. For instance, Cash et al. used cryo-EM complemented with HDXMS and enzymatic assays to fully resolve the structure of the P-Rex1 IP4P domain [39]. Another study that uses an integrative approach is from Su et al., where they used biochemical and biophysical analyses coupled with cryo-EM to fully characterise the Ebola virus nucleoprotein [40]. Furthermore, other characterisation methods not discussed here can be employed to detect PPIs. Miura [41] and Carter et al. [11] provided a detailed explanation and comparison of such methodologies.

### Molecular interactions involved in PPIs

There are different forces and mechanisms that affect PPIs and their formation, including hydrogen bonding [42–43], van der Waals interactions [44], hydrophobic interactions, and electrostatic forces. This section will provide an overview on the role of molecular interactions on PPIs and the mechanisms of protein–protein complex formation.

PPIs have to be specific enough for a particular protein to be able to recognize and interact with another partner protein among hundreds or thousands of other biomolecules [45]. Consequently, it was hypothesised that a long-range electrostatic guide or force is involved in bringing molecules together to interact non-covalently in vivo [46]. This is backed up by the fact that an electrostatic interaction between two molecules at 10 Å is around 1 kJ/mol, which is much higher than any other force at such a distance [47]. Long-range electrostatic interactions are affected by the net charges of the protein, i.e., proteins with a different net charge are electrostatically attracted to each other. In general, protein–protein complexes can be either composed of identical monomers, termed homooligomeric complexes, or non-identical ones, termed heterooligomeric complexes. With respect to heterooligomeric complexes, the monomers almost always have a different net charge, and thus are electrostatically attracted to each other. On the other hand, the identical monomers in homooligomeric complexes have identical net charges, and thus, in theory, long-range electrostatic forces oppose their attraction. As a result, the actual interaction in close proximal proteins is not governed by the net charge but by different localised charges on the accessible surface residues in the protein monomers. This creates a delicate balance between the interface interaction and the desolvation energy, which affects the binding free energy.

After binding occurs, ionisation changes can be induced in the protein molecules due to proton uptake and release. Therefore, these events are strongly affected by the pH value and the ionic strength. Variation of the pH value or the ionic strength can result in substantial binding free energy changes [48] or changes in the binding preference [49], producing two different modes of binding: pH-dependent and salt-dependent binding mechanisms. In a pH-dependent binding mechanism, an overall proton is either released or taken up during the protein interaction due to the binding-induced p*K*_a_ shift of acidic or basic amino acids present at the complex interface. Due to this shift, the interface amino acids experience either a significant desolvation energy, where there is a disruption in the residue charge–water interaction, resulting in water exclusion, and thus a hydrophobic effect [50], or an interaction in the complex formation. An example of the pH-dependence is β-lactoglobulin. The protein forms a dimer at a low pH value, while it is a tetramer at a higher pH value [51]. In a salt-dependent binding mechanism, the binding interaction occurs due to the changes in the solvent exposure of the charges in the contact residues before and after binding. This effect is termed the desalting effect [52] and is comparable to desolvation. This effect is also dependent on the charge–charge interactions of the complex as different charges might alter the ion distribution in the solvent, changing the ion interactions. On the other hand, an example of the ionic strength dependence is β-lactamase and its protein inhibitor BLIP, where binding decreases significantly as the salt concentration increases [53].

Chen et al. analysed the structural and thermodynamic data of 113 heterodimeric complexes and discussed the correlation between binding affinity and amount of surface area buried at the interface [54]. The authors determined this relationship by plotting the measured dissociation constant *K*_d_ against the buried surface area from the complex formation. The smallest complex studied was the transthyretin complex (2ROY), burying 381 Å^2^, whereas the largest complex, the SidM/DrrA-Rab1 complex (2WWX) has 3393 Å^2^ buried. They indicated that there is a trend between the buried interfacial surface area and the binding energy, where increasing the surface area increases the binding energy and thus *K*_d_. The authors also observed that the buried surface area has a high level of hydrophobicity, an average of 60% across all complexes studied. They also observed that there is no direct relationship between hydrophobic, aliphatic, polar charged, and polar uncharged, i.e., the nature of the residues on the surface area buried. On analysing the free energy per unit surface area buried, they show that the surface energy density is greater for smaller complexes (less than 2000 Å^2^ buried) than for larger ones (above 2000 Å^2^ buried). This is particularly important as, for smaller complexes, the interface has a high energetic contribution. Subsequently, any changes (i.e. mutations) in the interface residues have a higher energy contribution in smaller complexes than in larger complexes [54].

Complexes can be classified as either obligate, where the monomers are either unstable or/and non-functional when isolated or in solution, or non-obligate, where the monomers are stable. In obligate complexes, the interface is characterised by hydrophobic and aromatic residues while in non-obligate, the residues are more polar and charged, with the interface area being smaller [55] and containing more hydrogen bonds [56]. PPIs can also be differentiated by either being transient, meaning that the complex associates and dissociates in vivo, or permanent, meaning that the interaction is very stable and that the complex is only stable as an oligomer. Obligate interactions are normally permanent, while non-obligate interactions can be either [57].

The majority of proteins are found in complexes, with most of the complexes being homooligomeric complexes [58]. In fact, an analysis done in the BRENDA enzyme database [59] shows that there are more homooligomeric complexes than expected [60]. PPIs in homooligomeric complexes are usually difficult to predict using computational measures because the methods normally neglect self-interactions. The advantages of homooligomeric complexes over monomer proteins include an increase in the diversity of the functions [61], allosteric regulation [62], protection against denaturation [63], and the oligomers being able to form without increasing the genome size. It was determined that the interactions between two identical but randomly chosen surfaces are stronger than those between different surfaces of the same size [64]. Moreover, interactions decrease with a decreasing sequence identity [65], and the binding in isologous interfaces is more conserved than in non-isologous interfaces [66]. Furthermore, homooligomeric complexes have more disorder regions than heterooligomeric complexes, and thereby an increasing allosteric regulation [67].

Jayashree et al. [68] analysed datasets of 45 transient protein–protein complex structures and analysed the amino acid propensities at the protein–protein interface. They discussed that a large portion of the amino acids present at the interface (as large as 75%) are involved in so-called bifurcated interactions, where residues take part in both inter- and intraprotein interactions simultaneously. Both hydrophobic residues, such as leucine, phenylalanine, tryptophan, and methionine, as well as polar residues, such as aspartate, glutamate, histidine, and arginine, tend to be part of bifurcated interactions. On the other hand, glutamine and lysine were the only amino acids that tend to not take part in such interactions but have a high propensity to form interprotein interactions. In contrast, serine, threonine, asparagine (polar uncharged), alanine, valine, isoleucine (hydrophobic), cysteine, proline, and glycine are less likely to form interprotein interactions and bifurcated interactions. The binding mode in homooligomeric complexes can be due to either the interaction of identical amino acid residues or nonidentical amino acid residues on the complex interface, the latter having a similar approach to the heterooligomeric complex residues [69]. Some proteins can form both homooligomeric complexes and heterooligomeric complexes. Examples include integrin αIIb and β3 [70] as well as mammalian lipin isoforms, proteins that make up the phosphatidic acid phosphatase family [71], where lipin 1 can form stable homooligomeric complexes and heterooligomeric complexes with lipins 2 and 3.

**Figure 2:**
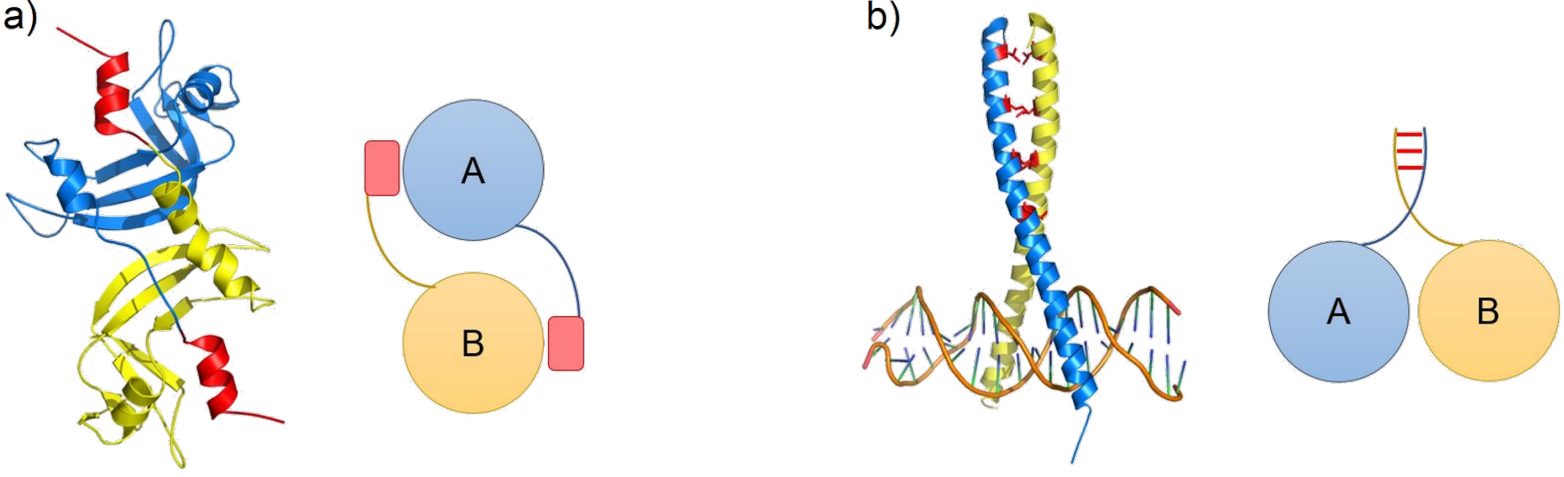
Mechanisms of homooligomeric complex formations. a) Domain swapping of RNase A (PDB: 1A2W) [73]. The swapped regions are shown in red. b) Leucine zipper of GCN4 (PDB: 1YSA) [74]. Interacting leucine residues are shown in red. Figure adapted from Hashimoto et al. [77].

Examples of homooligomeric complex formation include domain swapping and the formation of leucine zippers. In domain swapping, a region from the monomer interacts with the adjacent protein partner, forming the protein–protein interface. Most of the times, the interacting region is present on either the N- or C-terminus, although any part of the structure can produce this interaction [72]. An example for a protein that undergoes this mechanism is RNase A, where swapping the N and C termini results in complexes composed of either two monomers or more (Figure 2a) [73]. The leucine zipper is found in proteins as an interaction between domains in the form of an α-helical coiled coil structure. The common motif has heptad repeats in the format (abcdefg)*_n_*, where the ‘a’ and ‘d’ positions are hydrophobic residues (with leucine being in the d position most of the times). These residues interact to form the coiled coil structure. Different residues at the ‘a’ and ‘d’ positions give rise to different oligomeric states, with the yeast transcription factor GCN4 being a perfect example of this. GCN4 has isoleucine as ‘a’ and leucine as ‘d’ and forms a dimer (Figure 2b). When isoleucine and leucine are swapped via mutagenesis, a tetramer is produced [74]. This study also shows that the complex formation and PPIs are very susceptible to amino acid substitutions and insertions/deletions. In fact, it was observed that when an amino acid is substituted by a more hydrophobic residue with longer side chain groups, such as phenylalanine and tryptophan, the equilibrium between the monomer and the oligomer formation is shifted towards the latter [75–76].

### Examples of PPIs within higher-order protein complexes

PPIs are responsible for the assembly of large protein complexes, such as capsid proteins in viruses and protein containers. This section will give a brief overview on how such interactions can give rise to these complex molecules.

#### Capsid viruses

The infectivity in viruses is dependent on the correct assembly of viral capsids and surface proteins. A virus is made up of a genome enclosed by a number of copies of proteins to form a shell known as a capsid [78]. Some viruses also have lipid envelopes containing glycoproteins, which interact with the host cell membrane to facilitate the viral entry into the cell [79]. The simplest explanation for viral capsid assembly is protein assembly units colliding (following Brownian motion) in a perfect geometry to form the capsid irreversibly [80]. However, this approach is too simplistic and does not take into consideration kinetic traps where capsid formation cannot be completed due to a lack of assembly units. As a result, the use of assembly simulations and mathematical calculations shows three generalisations [81]. Firstly, errors and kinetic traps are minimised by weak interactions of the assembly units. The weak interactions result in nucleation, the second generalisation, where the initiation of capsid formation is minimised. This in return reduces the kinetic trap. Finally, the initial capsid formation is limited due to the time required for an intermediate steady state for the consequent assembly. These mathematical calculations and generalisations, however, do not take into consideration biological scaffolds, such as nucleic acids, which can facilitate or disrupt the capsid formation. This theoretical approach is backed up by experimental results from hepatitis B virus (HBV), where the homodimeric core protein assembles in vitro without any biological scaffold [82]. Nonetheless, HBV is a relatively simple system where the viral capsid is only made up of the homodimer [83]. In more complicated systems, such as in cowpea chlorotic mottle virus (CCMV), the viral capsid is made up of different assembly units. In vivo, viral nucleic acids may serve as biological scaffold to attract free assembly units [84] and organise on the surface [85]. Assembly with viral RNA present can be modelled by the McGhee–von Hippel model of nonspecific protein binding to a surface [86]. In this model, the association with nucleic acids, quantified by *K*_NA_, is dependent on the cooperativity coefficient ω, which is based on the protein–protein association constant. A ω value of 1 represents no cooperativity, whereas as a value greater than 1000 represents a high cooperativity, with the assembly occurring in two steps. CCMV shows a low cooperativity, and thus assembles gradually [87]. In contrast, HBV binds to RNA with a high cooperativity, resulting in a quantified assembly [88]. These results show that a nucleic acid scaffold aids the assembly by concentrating assembly units and providing additional association energy. Some viral capsids can self-assemble around other scaffolds that mimic the viral genome in charge and size [89] or can also assemble without any scaffold to form empty capsid containers [90]. CCMV was the first icosahedral viral capsid to be disassembled and reassembled in vitro [91]. The CCMV capsid can be reassembled into empty capsids as well as into different conformations, such as disks, rods, tubes, and multiwalled capsids by using different scaffolds [92]. In these cases, it has been shown that the CCMV capsid formation is driven by electrostatic interactions governed by the pH value and the ionic strength [93]. Here, the positive N terminus of the assembly unit interacts with the negatively charged scaffold and drives the self-assembly process [94].

**Figure 3:**
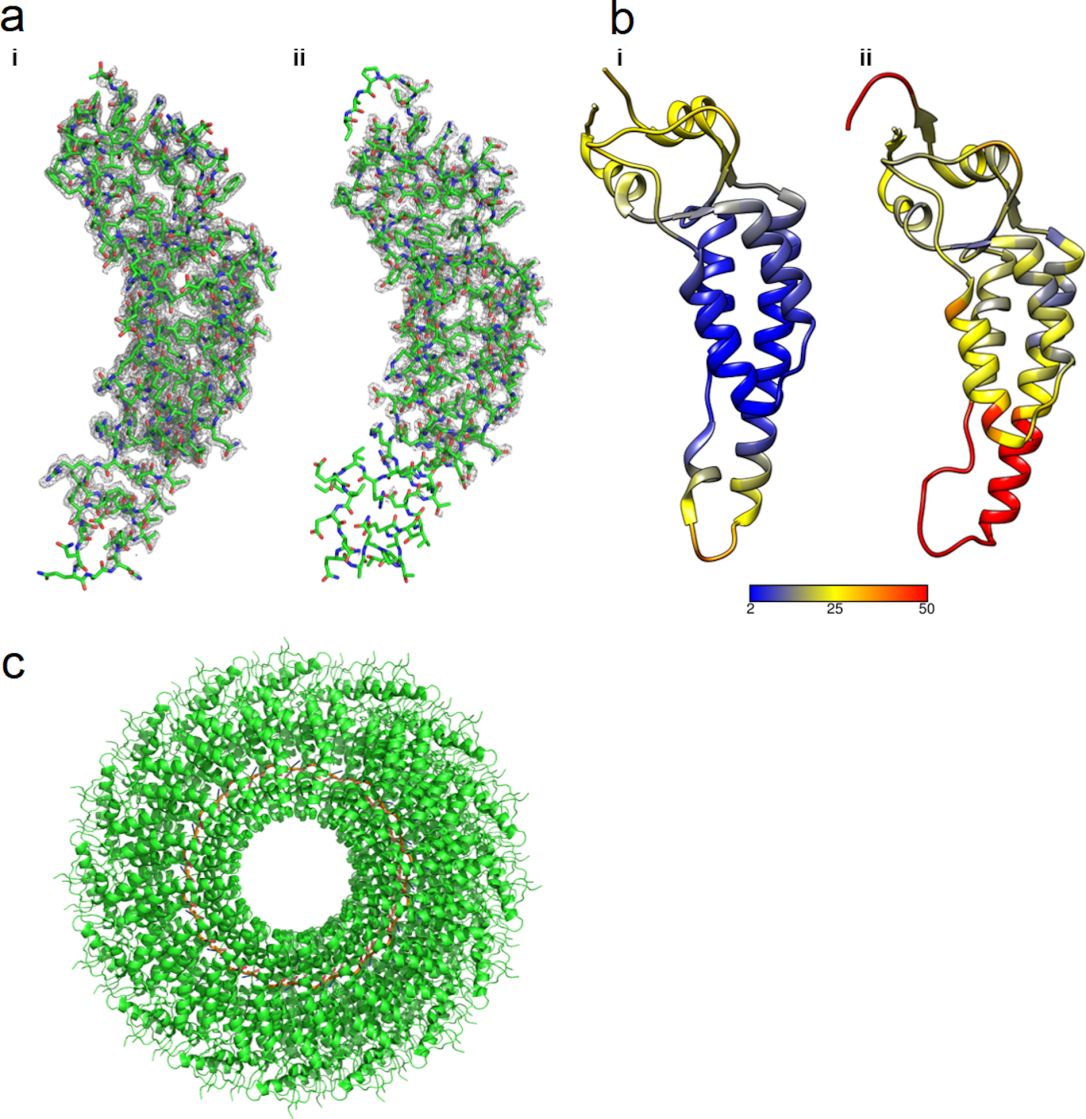
Parts a) and b) represent the electron densities and B-factors of TMV, respectively. The cryo-EM structure of TMV at 1.9 Å, PDB: 6SAE, EMD-10129 [95] is represented by i, whereas the X-ray structure of TMV at 2.45 Å, PDB: 1EI7 [96] is represented by ii, part c) shows the assembly of TMV [95]. The cryo-EM structure was able to elucidate loop regions that were not previously resolved in the X-ray structure.

The advent of cryo-EM technology has enabled the characterisation of more virus structures at a higher resolution. An example includes the tobacco mosaic virus, for which the structure has been characterised by both X-ray crystallography and cryo-EM. In fact, herein, the resolution obtained by cryo-EM (1.9 Å, PDB: 6SAE, EMD-10129 Figure 3a [95]) surpassed that obtained by X-ray crystallography (2.45 Å, PDB: 1EI7, Figure 3b [96]), showing the importance of cryo-EM in elucidating viral capsids. Other recent high-resolution cryo-EM viral capsid structures include human enterovirus D68 (2.17 Å, PDB: 6CSG, EMD-7599 [97]), rhinovirus B14 (2.26 Å, PDB: 5W3M, EMD-8762 [98]), and echovirus E11 (2.34 Å, PDB: 6LA3, EMD-0854 [99]).

#### Protein containers

**Figure 4:**
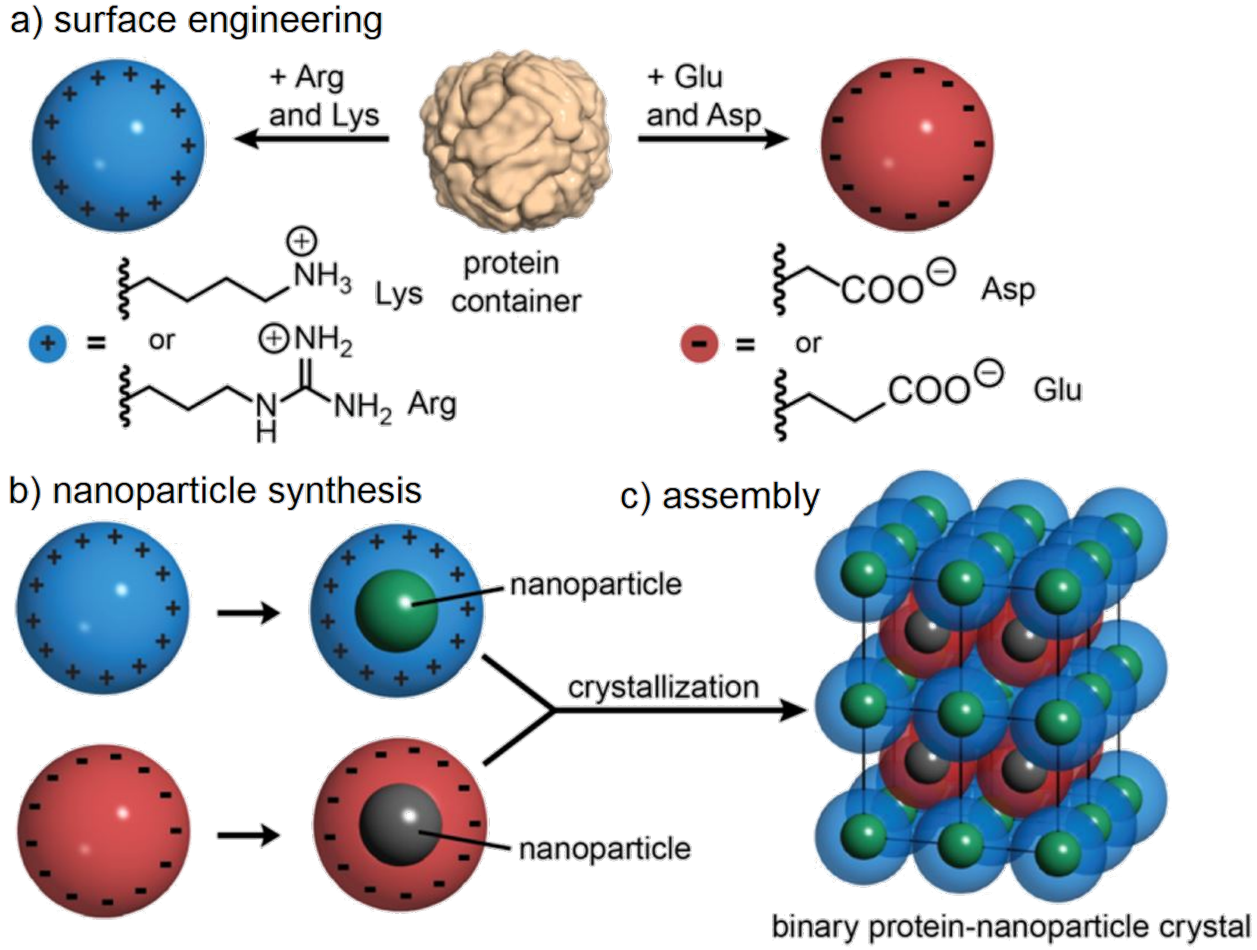
Schematic of the general assembly of charged protein containers to form binary nanoparticle superlattices. a) Surface engineering of the protein container to yield either positively (right) or negatively (left) charged containers. b) Nanoparticle synthesis. The different nanoparticles are illustrated in different colours. c) Self-assembly of the different charged protein containers to form a highly ordered three-dimensional superlattice. Figure reprinted with permission from *J. Am. Chem. Soc.* **2016**, *138*, 12731–12734 [103]. Copyright 2016 American Chemical Society.

Some non-viral proteins spontaneously self-assemble to form capsid structures (Figure 4). Examples include ferritin and lumazine synthase. Apart from their biological role, these proteins can be employed as containers in delivery vehicles [100], reaction vessels [101], and bioimaging agents [102]. These containers can also be modified to produce interactions between one container to another, and thus creating building blocks for nanoparticle assembly. Künzle et al. showed this by using ferritin [103]. Ferritin can be modified via mutagenesis to produce containers that contain a highly positively or negatively charged exterior surface. Interestingly, these mutations did not impede the formation of the capsid-like container and the mutated proteins assembled into containers in vitro. These highly charged variants were then used to form a binary three-dimensional assembly analogous to inorganic salts. The structure of this assembly was determined to have a 1:1 stoichiometry, with a coordination number of 12. The advantage of such systems was discussed to be that each variant can have a different cargo while maintaining homogeneity delivered by the protein structure [103].

## Conclusion

The discovery of PPIs and their mechanisms is an important avenue for understanding how protein–protein complexes function within the cell. In this minireview, we outlined some of the common characterisation methods employed to detect PPIs and gave a brief account on the shortcomings. Understanding the limitations provides a clearer approach on how to use different methodologies in unison to study and gain a better comprehension of PPIs. This review also examined the current knowledge of the molecular interactions and mechanisms that govern PPIs and their role in protein–protein complexes. We provided an outline on the different complexes known and how they are assembled. A clear perspective and a deeper understanding of these mechanisms and the roles of the complexes is fundamental in proteomics, and the identification of PPIs has the potential to confer new drug targets for diseases, such as cancer. As a result, research involving PPIs is imperative for our biological knowledge and future aspects in medicine.

## References

[1] Hedin S G (1906). Biochem J.

[2] Miki Y, Iwabuchi E, Ono K, Sasano H, Ito K (2018). Int J Mol Sci.

[3] Oldfield III E C (2007). Rev Gastroenterol Disord.

[4] Peng H-P, Lee K H, Jian J-W, Yang A-S (2014). Proc Natl Acad Sci U S A.

[5] Karimizadeh E, Sharifi-Zarchi A, Nikaein H, Salehi S, Salamatian B, Elmi N, Gharibdoost F, Mahmoudi M (2019). BMC Med Genomics.

[6] Smith T J, Chase E, Schmidt T, Perry K L (2000). J Virol.

[7] Ivanov A A, Khuri F R, Fu H (2013). Trends Pharmacol Sci.

[8] Zinzalla G, Thurston D E (2009). Future Med Chem.

[9] Chandra N, Padiadpu J (2013). Expert Opin Drug Discovery.

[10] Sun P, Ma T, Zhang T, Zhu H, Zhang J, Liu Y, Ding J (2019). J Biol Chem.

[11] Carter R, Luchini A, Liotta L, Haymond A (2019). Curr Pathobiol Rep.

[12] Powell H R (2019). Crystallogr Rev.

[13] Kaplan M, Pinto C, Houben K, Baldus M (2016). Q Rev Biophys.

[14] Frank J (2017). Nat Protoc.

[15] McPherson A, Gavira J A (2014). Acta Crystallogr, Sect F: Struct Biol Commun.

[16] Tagari M, Newman R, Chagoyen M, Carazo J-M, Henrick K (2002). Trends Biochem Sci.

[R17] 17Yip, K. M. F. N., Chari A., Stark H., unpublished results.

[18] Lytvynenko I, Paternoga H, Thrun A, Balke A, Müller T A, Chiang C H, Nagler K, Tsaprailis G, Anders S, Bischofs I (2019). Cell.

[19] Gruene T, Wennmacher J T C, Zaubitzer C, Holstein J J, Heidler J, Fecteau-Lefebvre A, De Carlo S, Müller E, Goldie K N, Regeni I (2018). Angew Chem, Int Ed.

[20] Zhou H, Luo F, Luo Z, Li D, Liu C, Li X (2019). Anal Chem (Washington, DC, U S).

[21] Nannenga B L, Gonen T (2019). Nat Methods.

[22] Luchini A, Espina V, Liotta L A (2014). Nat Commun.

[23] Rappsilber J, Siniossoglou S, Hurt E C, Mann M (2000). Anal Chem (Washington, DC, U S).

[24] Masson G R, Burke J E, Ahn N G, Anand G S, Borchers C, Brier S, Bou-Assaf G M, Engen J R, Englander S W, Faber J (2019). Nat Methods.

[25] Johnson D T, Di Stefano L H, Jones L M (2019). J Biol Chem.

[26] Hvidt A, Linderstrøm-Lang K (1954). Biochim Biophys Acta.

[27] Myszka D (2006). Abstr Pap - Am Chem Soc.

[28] Omar N A S, Fen Y W (2018). Sens Rev.

[29] Bai Y, Meyerkord C, Fu H (2015). Detecting Protein-Protein Interactions by Gel Filtration Chromatography. Protein-Protein Interactions.

[30] Velazquez-Campoy A, Leavitt S A, Freire E, Meyerkord C, Fu H (2015). Characterization of Protein-Protein Interactions by Isothermal Titration Calorimetry. Protein-Protein Interactions.

[31] Prozeller D, Morsbach S, Landfester K (2019). Nanoscale.

[32] Zong H, Wang X, Mu X, Wang J, Sun M (2019). Chem Rec.

[33] Jerabek-Willemsen M, André T, Wanner R, Roth H M, Duhr S, Baaske P, Breitsprecher D (2014). J Mol Struct.

[34] Hussack G, Baral T N, Baardsnes J, van Faassen H, Raphael S, Henry K A, Zhang J, MacKenzie C R (2017). Front Immunol.

[35] Kimple A J, Muller R E, Siderovski D P, Willard F S (2010). A Capture Coupling Method for the Covalent Immobilization of Hexahistidine Tagged Proteins for Surface Plasmon Resonance. Surface Plasmon Resonance: Methods and Protocols.

[36] Patching S G (2014). Biochim Biophys Acta, Biomembr.

[37] Ofran Y, Rost B (2007). PLoS Comput Biol.

[38] Keskin O, Tuncbag N, Gursoy A (2016). Chem Rev.

[39] Cash J N, Urata S, Li S, Ravala S K, Avramova L V, Shost M D, Gutkind J S, Tesmer J J G, Cianfrocco M A (2019). Sci Adv.

[40] Su Z, Wu C, Shi L, Luthra P, Pintilie G D, Johnson B, Porter J R, Ge P, Chen M, Liu G (2018). Cell.

[41] Miura K (2018). Protein Pept Lett.

[42] Sawyer N, Watkins A M, Arora P S (2017). Acc Chem Res.

[43] Kortemme T, Morozov A V, Baker D (2003). J Mol Biol.

[44] Nilofer C, Sukhwal A, Mohanapriya A, Kangueane P (2017). Bioinformation.

[45] Carbonell P, Nussinov R, del Sol A (2009). Proteomics.

[46] Cheng Y, Holst M J, McCammon J A (2009). Pac Symp Biocomput.

[47] Zhang Z, Witham S, Alexov E (2011). Phys Biol.

[48] Sprague E R, Martin W L, Bjorkman P J (2004). J Biol Chem.

[49] Gramberg T, Soilleux E, Fisch T, Lalor P F, Hofmann H, Wheeldon S, Cotterill A, Wegele A, Winkler T, Adams D H (2008). Virology.

[50] Prasad Bahadur R, Chakrabarti P, Rodier F, Janin J (2004). J Mol Biol.

[51] Sakurai K, Oobatake M, Goto Y (2001). Protein Sci.

[52] Bertonati C, Honig B, Alexov E (2007). Biophys J.

[53] Albeck S, Schreiber G (1999). Biochemistry.

[54] Chen J, Sawyer N, Regan L (2013). Protein Sci.

[55] Bahadur R P, Chakrabarti P, Rodier F, Janin J (2003). Proteins: Struct, Funct, Genet.

[56] Zhanhua C, Gan J G-K, lei L, Sakharkar M K, Kangueane P (2005). Bioinformation.

[57] Nooren I M A, Thornton J M (2003). EMBO J.

[58] Cornish-Bowden A J, Koshland D E (1971). J Biol Chem.

[59] Schomburg I, Chang A, Ebeling C, Gremse M, Heldt C, Huhn G, Schomburg D (2004). Nucleic Acids Res.

[60] Ispolatov I, Yuryev A, Mazo I, Maslov S (2005). Nucleic Acids Res.

[61] Woodcock J M, Murphy J, Stomski F C, Berndt M C, Lopez A F (2003). J Biol Chem.

[62] Changeux J P, Edelstein S J (2005). Science.

[63] Goodsell D S, Olson A J (2000). Annu Rev Biophys Biomol Struct.

[64] Lukatsky D B, Shakhnovich B E, Mintseris J, Shakhnovich E I (2007). J Mol Biol.

[65] Wright C F, Teichmann S A, Clarke J, Dobson C M (2005). Nature.

[66] Dayhoff J E, Shoemaker B A, Bryant S H, Panchenko A R (2010). J Mol Biol.

[67] Simon S M, Sousa F J R, Mohana-Borges R, Walker G C (2008). Proc Natl Acad Sci U S A.

[68] Jayashree S, Murugavel P, Sowdhamini R, Srinivasan N (2019). Biol Direct.

[69] Talley K, Ng C, Shoppell M, Kundrotas P, Alexov E (2008). PMC Biophys.

[70] Zhu H, Metcalf D G, Streu C N, Billings P C, DeGrado W F, Bennett J S (2010). J Mol Biol.

[71] Liu G-H, Qu J, Carmack A E, Kim H B, Chen C, Ren H, Morris A J, Finck B N, Harris T E (2010). Biochem J.

[72] Bennett M J, Choe S, Eisenberg D (1994). Proc Natl Acad Sci U S A.

[73] Liu Y, Gotte G, Libonati M, Eisenberg D (2001). Nat Struct Biol.

[74] Harbury P B, Zhang T, Kim P S, Alber T (1993). Science.

[75] Nishi H, Ota M (2010). Proteins: Struct, Funct, Bioinf.

[76] Grueninger D, Treiber N, Ziegler M O P, Koetter J W A, Schulze M-S, Schulz G E (2008). Science.

[77] Hashimoto K, Nishi H, Bryant S, Panchenko A R (2011). Phys Biol.

[78] Crick F H C, Watson J D (1956). Nature.

[79] Koch B, Dolnik O, Büttner S, Becker S, Geiger H, Baer P (2017). Nephrol, Dial, Transplant.

[80] Hemberg M, Yaliraki S N, Barahona M (2006). Biophys J.

[81] Rapaport D C (2008). Phys Rev Lett.

[82] Stray S J, Bourne C R, Punna S, Lewis W G, Finn M G, Zlotnick A (2005). Proc Natl Acad Sci U S A.

[83] Zlotnick A, Johnson J M, Wingfield P W, Stahl S J, Endres D (1999). Biochemistry.

[84] Datta S A K, Curtis J E, Ratcliff W, Clark P K, Crist R M, Lebowitz J, Krueger S, Rein A (2007). J Mol Biol.

[85] Hagan M F (2009). J Chem Phys.

[86] McGhee J D, von Hippel P H (1974). J Mol Biol.

[87] Johnson J M, Willits D A, Young M J, Zlotnick A (2004). J Mol Biol.

[88] Porterfield J Z, Dhason M S, Loeb D D, Nassal M, Stray S J, Zlotnick A (2010). J Virol.

[89] Chen C, Daniel M-C, Quinkert Z T, De M, Stein B, Bowman V D, Chipman P R, Rotello V M, Kao C C, Dragnea B (2006). Nano Lett.

[90] Bancroft J B, Bracker C E, Wagner G W (1969). Virology.

[91] Bancroft J B, Hills G J, Markham R (1967). Virology.

[92] Durán-Meza A L, Escamilla-Ruiz M I, Segovia-González X F, Villagrana-Escareño M V, Vega-Acosta J R, Ruiz-Garcia J (2020). Molecules.

[93] Lavelle L, Gingery M, Phillips M, Gelbart W M, Knobler C M, Cadena-Nava R D, Vega-Acosta J R, Pinedo-Torres L A, Ruiz-Garcia J (2009). J Phys Chem B.

[94] Vega-Acosta J R, Cadena-Nava R D, Gelbart W M, Knobler C M, Ruiz-García J (2014). J Phys Chem B.

[95] Weis F, Beckers M, von der Hocht I, Sachse C (2019). EMBO Rep.

[96] Bhyravbhatla B, Watowich S J, Caspar D L D (1998). Biophys J.

[97] Liu Y, Sheng J, van Vliet A L W, Buda G, van Kuppeveld F J M, Rossmann M G (2018). Proc Natl Acad Sci U S A.

[98] Dong Y, Liu Y, Jiang W, Smith T J, Xu Z, Rossmann M G (2017). Proc Natl Acad Sci U S A.

[99] Niu S, Liu C, Liu C, Liu S, Song Y, Zhang Y, Tian W, Zhao X, Wang P, Gao G F (2020). Chin Sci Bull.

[100] Stephanopoulos N, Tong G J, Hsiao S C, Francis M B (2010). ACS Nano.

[101] Bode S A, Minten I J, Nolte R J M, Cornelissen J J L M (2011). Nanoscale.

[102] Liepold L O, Abedin M J, Buckhouse E D, Frank J A, Young M J, Douglas T (2009). Nano Lett.

[103] Künzle M, Eckert T, Beck T (2016). J Am Chem Soc.

